# Modeling of Future COVID-19 Cases, Hospitalizations, and Deaths, by Vaccination Rates and Nonpharmaceutical Intervention Scenarios — United States, April–September 2021

**DOI:** 10.15585/mmwr.mm7019e3

**Published:** 2021-05-14

**Authors:** Rebecca K. Borchering, Cécile Viboud, Emily Howerton, Claire P. Smith, Shaun Truelove, Michael C. Runge, Nicholas G. Reich, Lucie Contamin, John Levander, Jessica Salerno, Wilbert van Panhuis, Matt Kinsey, Kate Tallaksen, R. Freddy Obrecht, Laura Asher, Cash Costello, Michael Kelbaugh, Shelby Wilson, Lauren Shin, Molly E. Gallagher, Luke C. Mullany, Kaitlin Rainwater-Lovett, Joseph C. Lemaitre, Juan Dent, Kyra H. Grantz, Joshua Kaminsky, Stephen A. Lauer, Elizabeth C. Lee, Hannah R. Meredith, Javier Perez-Saez, Lindsay T. Keegan, Dean Karlen, Matteo Chinazzi, Jessica T. Davis, Kunpeng Mu, Xinyue Xiong, Ana Pastore y Piontti, Alessandro Vespignani, Ajitesh Srivastava, Przemyslaw Porebski, Srinivasan Venkatramanan, Aniruddha Adiga, Bryan Lewis, Brian Klahn, Joseph Outten, James Schlitt, Patrick Corbett, Pyrros Alexander Telionis, Lijing Wang, Akhil Sai Peddireddy, Benjamin Hurt, Jiangzhuo Chen, Anil Vullikanti, Madhav Marathe, Jessica M. Healy, Rachel B. Slayton, Matthew Biggerstaff, Michael A. Johansson, Katriona Shea, Justin Lessler

**Affiliations:** ^1^The Pennsylvania State University, State College, Pennsylvania; ^2^Fogarty International Center, National Institutes of Health, Bethesda, Maryland; ^3^Johns Hopkins Bloomberg School of Public Health, Baltimore, Maryland; ^4^U.S. Geological Survey, Laurel, Maryland; ^5^University of Massachusetts Amherst, Amherst, Massachusetts; ^6^University of Pittsburgh, Pittsburgh, Pennsylvania; ^7^Johns Hopkins University Applied Physics Laboratories, Laurel, Maryland; ^8^École polytechnique fédérale de Lausanne, Lausanne, Switzerland; ^9^University of Utah, Salt Lake City, Utah; ^10^University of Victoria, Victoria, British Columbia, Canada; ^11^Northeastern University, Boston, Massachusetts; ^12^University of Southern California, Los Angeles, California; ^13^University of Virginia, Charlottesville, Virginia; ^14^CDC COVID-19 Response Team.

After a period of rapidly declining U.S. COVID-19 incidence during January–March 2021, increases occurred in several jurisdictions (*1*,*2*) despite the rapid rollout of a large-scale vaccination program. This increase coincided with the spread of more transmissible variants of SARS-CoV-2, the virus that causes COVID-19, including B.1.1.7 (*1*,*3*) and relaxation of COVID-19 prevention strategies such as those for businesses, large-scale gatherings, and educational activities. To provide long-term projections of potential trends in COVID-19 cases, hospitalizations, and deaths, COVID-19 Scenario Modeling Hub teams used a multiple-model approach comprising six models to assess the potential course of COVID-19 in the United States across four scenarios with different vaccination coverage rates and effectiveness estimates and strength and implementation of nonpharmaceutical interventions (NPIs) (public health policies, such as physical distancing and masking) over a 6-month period (April–September 2021) using data available through March 27, 2021 (*4*). Among the four scenarios, an accelerated decline in NPI adherence (which encapsulates NPI mandates and population behavior) was shown to undermine vaccination-related gains over the subsequent 2–3 months and, in combination with increased transmissibility of new variants, could lead to surges in cases, hospitalizations, and deaths. A sharp decline in cases was projected by July 2021, with a faster decline in the high-vaccination scenarios. High vaccination rates and compliance with public health prevention measures are essential to control the COVID-19 pandemic and to prevent surges in hospitalizations and deaths in the coming months.

Following previous short-term disease forecasting efforts, the COVID-19 Scenario Modeling Hub (*4*) convened six modeling teams in an open call to provide long-term, 6-month (April–September 2021) COVID-19 projections in the United States using data available through March 27, 2021 (*2*,*5*). Teams each developed a model to project weekly reported cases, hospitalizations, and deaths, both nationally and by jurisdiction (50 states and the District of Columbia), for each scenario, using data from the Johns Hopkins Center for Systems Science and Engineering Coronavirus Resource Center and federal databases (*2*,*5*). Four scenarios were considered in each model: high vaccination with moderate NPI use, high vaccination with low NPI use, low vaccination with moderate NPI use, and low vaccination with low NPI use (*4*) (Table) Vaccination scenarios took into account vaccine effectiveness (VE), weekly state-specific data on COVID-19 vaccination rates, and age- and risk-specific vaccine prioritization (e.g., older adults and health care workers); VE estimates were based on protection against clinical disease in randomized clinical trials^§^; parameters for effectiveness against infection and transmission were determined by each modeling team (*4*). For each NPI scenario, teams estimated a level of NPI adherence in March 2021 and then implemented a linear decrease of that level beginning in April to be 50% or 80% lower in September 2021. All scenarios included the spread of the B.1.1.7 variant, with the assumption that it was 50% more transmissible than were previously circulating SARS-CoV-2 variants (*3*,*4*). Individual modeling teams provided probabilistic projections for each future week, characterizing uncertainty with quantiles. These were combined into an ensemble for each scenario, outcome, week, and location by using the median across teams for each quantile (*4*,*6*). The individual models differed substantially in structure and design (*4*), but all accounted for age groups, enabling prioritization of vaccination based on federal and state guidelines.

**TABLE:** COVID-19 projection scenarios* **—** United States, March 27**–**September 25, 2021

Vaccination and NPIs	Moderate NPI use; moderate reduction in NPI	Low NPI use; high reduction in NPI
**High vaccination (high VE, administration, and vaccine coverage)**		
Moderna/Pfizer (2 doses)	75%/95% VE against symptoms^†^ 50M 1st doses administered monthly during Apr–Sep 2021^§^	75%/95% VE against symptoms^†^ 50M 1st doses administered monthly during Apr–Sep 2021^§^
Johnson & Johnson (1 dose)	70% VE against symptoms^†^ 10–20M doses administered monthly (Apr: 10M, May: 15M, Jun–Sep: 20M)^§^	70% VE against symptoms^†^ 10–20M doses administered monthly (Apr: 10M, May: 15M, June–Sep: 20M)^§^
Vaccination coverage per group^¶^	Maximum = 90%	Maximum = 90%
**NPIs**	Estimated NPI levels in Mar 2021 are gradually reduced by 50% during Apr–Sep 2021	Estimated NPI levels in Mar 2021 are gradually reduced by 80% during Apr–Sep 2021
**Low vaccination (low VE, administration, and vaccine coverage)**		
Moderna/Pfizer (2 doses)	50%/85% VE against symptoms^†^ 45M 1st doses administered monthly during Apr–Sep 2021^§^	50%/85% VE against symptoms^†^ 45M 1st doses administered monthly during Apr–Sep 2021^§^
Johnson & Johnson (1 dose)	60% VE against symptoms^†^ 5M doses administered monthly during Apr–Sep 2021^§^	60% VE against symptoms^†^ 5M doses administered monthly during Apr–Sep 2021^§^
Vaccination coverage per group^¶^	Maximum = 75%	Maximum = 75%
**NPIs**	Estimated NPI levels in Mar 2021 are gradually reduced by 50% during Apr–Sep 2021	Estimated NPI levels in Mar 2021 are gradually reduced by 80% during Apr–Sep 2021

**Abbreviations:** M = million; NPI = nonpharmaceutical interventions; VE = vaccine effectiveness.

* Scenarios were defined to control for uncertainty in two specific factors: vaccination and adherence to NPIs with high/moderate and low levels for each. All scenarios included the B.1.1.7 variant and assumed that it was 50% more transmissible than previously circulating SARS-CoV-2 variants. All other transmission and outcome assumptions were decided by the six modeling teams.

^†^ VE is defined as vaccine effectiveness against symptomatic disease 2 weeks after administration, based on clinical trials. For 2-dose vaccines, the first VE represents protection 2 weeks after the 1st dose. Assumptions about effectiveness and affects on other outcomes (e.g., infection, hospitalization, and death) were left to the discretion of individual teams. Five teams assumed that VE against infection was the same as VE against symptomatic disease, and one team assumed lower VE against infection; details on model structure and assumptions are available at MIDAS Network COVID-19 Scenario Modeling Hub. Accessed April 19, 2021. https://github.com/midas-network/covid19-scenario-modeling-hub

^§^ Vaccine doses reflect published manufacturing capacity estimates in the high vaccination scenarios and a continuation of the pace of vaccination observed at the end of March 2021 in the low vaccination scenarios.

^¶^ If the maximum level of vaccination specified (e.g., 75% or 90%) was reached in a population group during the projection period, models assume that no more vaccination occurs in that group. Past reported vaccine coverage up to March 27, 2021, can exceed these levels.

In all four scenarios, COVID-19 cases were projected to increase through May 2021 at the national level because of increased prevalence of the B.1.1.7 variant and decreased NPI mandates and compliance (Figure 1). A sharp decline in cases was projected by July 2021, with a faster decline in the high-vaccination scenarios. Increases in hospitalizations and deaths (Figure 1), although more moderate, were also projected. A peak of 7,000–11,100 weekly deaths nationwide was projected in May (range = 5,382–15,677, which includes the central 50% of the projected distributions for all scenarios in the ensemble). The larger increases in cases relative to hospitalizations and deaths were attributable to higher vaccination coverage among groups with higher risk for severe COVID-19.

**FIGURE 1:**
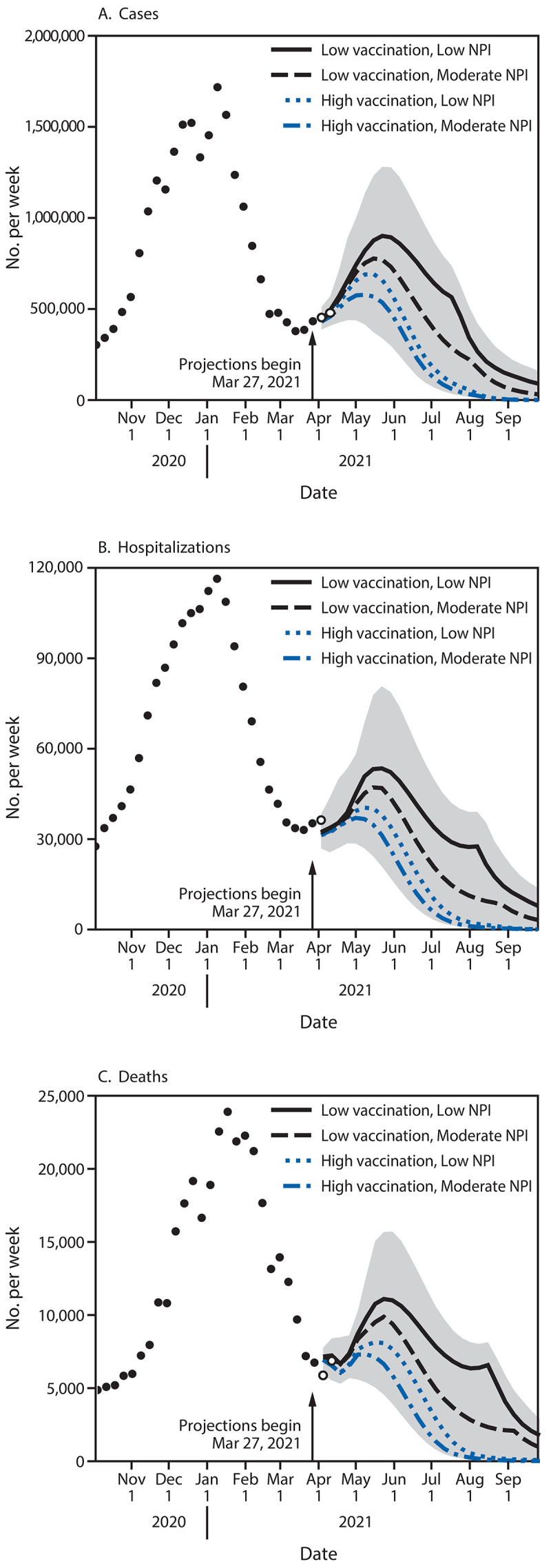
Weekly projections of reported numbers of cases (A), hospitalizations (B), and deaths (C)* under four scenarios representing different levels of vaccination and nonpharmaceutical intervention adherence — United States, March 27–September 25, 2021 **Abbreviation:** NPI = nonpharmaceutical intervention. * Historical data are shown as filled points, curves represent ensemble projections based on six models, and the grey area represents the maximum and minimum of the 50% projection intervals among all four scenarios. Vertical arrows represent the last date of observations used in the projections. Observations available after projections were made are shown as open points. Projection intervals are based on the 25th percentile of the more optimistic scenario (high vaccination and moderate NPI use) and the 75th percentile of the more pessimistic scenario (low vaccination and low NPI use). Ensemble projection curves represent the median of six median model projections, so they might not always appear smooth; the discontinuity in low vaccination scenario ensembles arises as two models project a late summer resurgence.

Moderate NPI use reduced cases and deaths in both the high and low vaccination scenarios, compared with low NPI use. The effect of maintaining moderate levels of NPI adherence was larger in the low vaccination scenarios, illustrating the counterbalance between and complementary effects of the two strategies (Figure 2). When low vaccination coverage was combined with low NPI adherence, cumulative cases, hospitalizations, and deaths were substantially higher compared with other scenarios. The largest differences among scenarios was in the cumulative excess percentage of hospitalizations. Differences in deaths were lower because many of the groups at highest risk were already vaccinated at the beginning of the projection window. Differences in cases were relatively small because in all scenarios a substantial number of new cases occurred.

**FIGURE 2:**
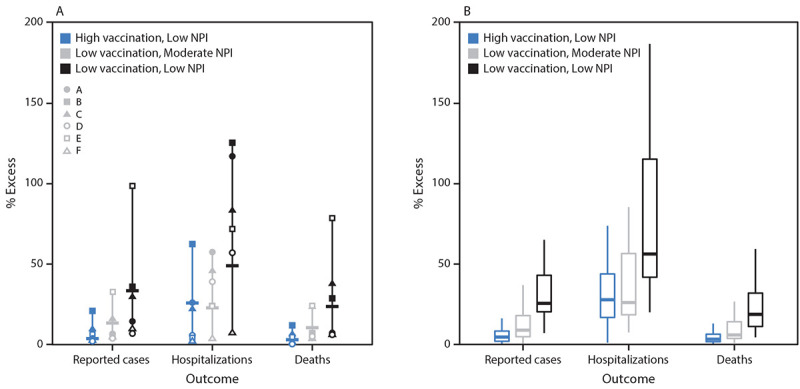
Excess percentage of reported cases, hospitalizations, and deaths projected to occur under scenarios with reduced vaccination coverage, nonpharmaceutical intervention adherence, or both, compared with the more optimistic scenario (high vaccination and moderate nonpharmaceutical intervention adherence),* nationally (A)^†^ and by state (B)^§^ — United States, March 27–September 25, 2021 **Abbreviation:** NPI = nonpharmaceutical intervention. * Cumulative estimates for the projection period March 27–September 25, 2021, are compared with the more optimistic scenario (high vaccination and moderate NPI). ^†^ National estimates represent the range of projections generated by the six contributing teams (symbols = individual models, dash = ensemble median). Individual models have been developed by six academic teams and are named JHU_IDD-CovidSP (A); JHUAPL-Bucky (B); Karlen-pypm (C); MOBS_NEU-GLEAM_COVID (D); USC-SIkJalpha (E); and UVA-adaptive (F). Details on model structure and assumptions are available at MIDAS Network COVID-19 Scenario Modeling Hub. Accessed April 19, 2021. https://github.com/midas-network/covid19-scenario-modeling-hub ^§^ Box plots represent the distribution of ensemble estimates in the 50 U.S. states and the District of Columbia. Boxes represent the interquartile range and the horizontal lines within each box represent the median. The whiskers extend to the most extreme data point that is no further from the box than 1.5 times the interquartile range.

Whereas the benefits of increased control measures varied substantially between models, the largest excess percentages in estimated effects for each model were consistently found in scenarios with the lowest NPI use and vaccination levels (Figure 2). Considerable range in state-specific projections was observed (Figure 2), suggesting that some states could reach levels of disease similar to those observed in late 2020 in scenarios with lower use of NPIs.

## Discussion

In this modeling study using data through March 27, 2021, COVID-19 cases were projected to increase nationally in April and peak in May 2021 in four assessed scenarios of vaccination coverage and NPI adherence. A moderate resurgence in deaths and hospitalizations was also projected during this period. Nationally, reported cases, hospitalization, and deaths are now decreasing or stable. However, transmission remains widespread and increased cases, hospitalizations, and deaths continue to be reported in some jurisdictions and, as this study indicates, the potential for future increases persists. Within each modeled scenario, substantial variation existed in the projected trajectory within individual states, potentially driven by the differences in the levels of population immunity, introduction and expansion of new variants, effectiveness of existing NPIs, and vaccine acceptance and coverage. Even moderate reductions in NPI adherence were shown to undermine vaccination-related gains during the subsequent 2–3 months; decreased NPI adherence, in combination with increased transmissibility of some new variants, was projected to lead to surges in hospitalizations and deaths. Based on these findings, public health messaging to encourage vaccination and use of effective NPIs is essential to control the COVID-19 pandemic and prevent increases in COVID-19–related hospitalizations and deaths in the coming months.

All contributing models attributed increased SARS-CoV-2 transmission in many parts of the United States to the relaxation of mitigation strategies and the increasing prevalence of more transmissible variants, although the relative contribution of each factor varied among models. The emergence of new variants has been associated with resurgence in cases, hospitalizations, and deaths in Europe, South Africa, Brazil, and India, requiring new restrictions to prevent local outbreaks. In the United States, B.1.1.7 and other variants of domestic and international origin were projected to drive continued increases in case counts in the coming months (*3*) and could negate recent gains in controlling SARS-CoV-2 transmission. This is consistent with the findings in this study, which indicate that local conditions and rapid establishment of emerging variants place many states at risk for high incidences of COVID-19 cases in the spring, potentially requiring implementation of increased control measures to limit SARS-CoV-2 spread.

This is the first multiple model effort to project long-term trajectories of COVID-19 in real-time in the United States under different epidemiologic scenarios. Model differences identified critical areas of uncertainty, including vaccine acceptance, adherence to recommended NPIs, prevalence of the B.1.1.7 variant, duration of immunity, and state-level NPI policies (*4*). These models can be updated in response to changing conditions through new scenarios, updated fitting or structural changes of individual models, and the addition of new models. In contrast to the results generated by the COVID-19 Forecasting Hub (*6*), the projections in this study are intended to bound plausible outbreak trajectories and should not be considered forecasts of the most likely outcome. These projections could be used for planning purposes (e.g., to estimate needs for COVID-19 treatments and hospital beds) and to guide public health efforts (e.g., to balance vaccination efforts with implementation of NPIs).

The findings in this report are subject to at least four limitations. First, considerable uncertainty is inherent when modeling the trajectory of COVID-19 over longer time frames (*7*,*8*). Whereas this analysis identifies a range of realistic uncertainty through well-defined scenarios and by combining multiple models, unforeseen events (e.g., a temporary pause in the use of a vaccine) could cause deviations that might not be reflected by the modeled scenarios (e.g., low and high vaccination). Second, only the B.1.1.7 variant was included in the scenarios given its increasing prevalence in the United States at the time modeling groups were convened and its increased transmissibility. The effect of B.1.1.7, as modeled, can be considered a proxy for more transmissible variants in general, but other emerging variants might have different effects. Third, the estimates are limited to six models based on existing data, and the models might not fully encompass the range of plausible trajectories. A larger number of models would better represent uncertainty in the epidemiology of COVID-19 (*8*). Finally, one approach to combining individual models and model-specific uncertainty into a single ensemble projection for each scenario was used (*9*). Different approaches to combining individual models into an ensemble changed the magnitude, but not the direction, of the expected impacts. Regardless of the approach used to generate the ensembles, they do not convey all potentially divergent trajectories that individual models project.

The rapid rollout of vaccination is having a positive impact on the COVID-19 pandemic in the United States and reported disease nationally during April has been on the lower end of the scenario projections to date. However, multiple jurisdictions have seen a resurgence of COVID-19 cases and others likely will if NPI adherence declines too rapidly. Increases in deaths and hospitalizations could be more moderate because of prioritization of vaccination groups at high risk for COVID-19 but are still expected, particularly in locations with pronounced increases in transmission earlier during the vaccine rollout. These modeled scenarios show that ongoing efforts to continue to increase vaccination coverage and maintain physical distancing, masking, isolation, and quarantine are warranted. As the COVID-19 pandemic evolves and more data become available regarding factors affecting outbreak dynamics, future projections from the COVID-19 Scenario Modeling Hub can provide new and improved insights for public health response (*10*).

**Summary**

**What is already known about this topic?**

Increases in COVID-19 cases in March and early April occurred despite a large-scale vaccination program. Increases coincided with the spread of SARS-CoV-2 variants and relaxation of nonpharmaceutical interventions (NPIs).

**What is added by this report?**

Data from six models indicate that with high vaccination coverage and moderate NPI adherence, hospitalizations and deaths will likely remain low nationally, with a sharp decline in cases projected by July 2021. Lower NPI adherence could lead to substantial increases in severe COVID-19 outcomes, even with improved vaccination coverage.

**What are the implications for public health practice?**

High vaccination coverage and compliance with NPIs are essential to control COVID-19 and prevent surges in hospitalizations and deaths in the coming months.

## Footnotes

* These authors contributed equally as first authors.

† These authors contributed equally as senior authors.

§ https://www.cdc.gov/mmwr/volumes/69/wr/mm695152e1.htm?s_cid=mm695152e1_w; https://www.cdc.gov/mmwr/volumes/69/wr/mm6950e2.htm?s_cid=mm6950e2_w; https://www.cdc.gov/mmwr/volumes/70/wr/mm7009e4.htm?s_cid=mm7009e4_w
